# Decolourization of 4-Chloro-2-Nitrophenol by a Soil Bacterium, *Bacillus subtilis* RKJ 700

**DOI:** 10.1371/journal.pone.0052012

**Published:** 2012-12-12

**Authors:** Pankaj Kumar Arora

**Affiliations:** Environmental Biotechnology, CSIR-Institute of Microbial Technology, Chandigarh, India; Imperial College London, United Kingdom

## Abstract

A 4-Chloro-2-nitrophenol (4C2NP) decolourizing strain RKJ 700 was isolated from soil collected from a pesticide contaminated site of India and identified as *Bacillus subtilis* on the basis of the 16S rRNA gene sequence analysis. *Bacillus subtilis* RKJ 700 decolourized 4C2NP up to concentration of 1.5 mM in the presence of additional carbon source. The degradation pathway of 4C2NP was studied and 4-chloro-2-aminophenol, 4-chloro-2-acetaminophenol and 5-chloro-2-methylbenzoxazole (5C2MBZ) were identified as metabolites by high performance liquid chromatography and gas chromatography-mass spectrometry. Resting cell studies showed that *Bacillus subtilis* RKJ 700 depleted 4C2NP completely with stoichiometric formation of 5C2MBZ. This is the first report of (i) the degradation of 4C2NP at high concentration (1.5 mM) and, (ii) the formation of 5C2MBZ by a soil bacterium.

## Introduction

4-Chloro-2-nitrophenol (4C2NP) is a highly toxic and recalcitrant compound, which is used for manufacturing of dyes, pesticides, drugs and chemicals [1]. Due to its widespread applications, agriculture soil and water resources, including groundwater and surface water, have become contaminated with 4C2NP. The contaminated ground water serves as the primary source of drinking water, thereby posing a serious environment and human health concern that must be addressed on the priority basis. This problem can be solved by developing new and safer methods for decontamination, which can be implemented for restoration of previously contaminated sites. Like most of the other chemical pollutants, 4C2NP can also be degraded by either the physico-chemical methods or with microbial degradation [1].

In the last decade, several reports have been published on the degradation of 4C2NP by physico-chemical methods [1]. Saritha et al. [2] used various advanced oxidation process to degrade 4C2NP and reported that UV or H_2_O_2_ was not capable to degrade 4C2NP. The combination of both i.e., UV with H_2_O_2_ increased the efficiency of the mineralization of 4C2NP. The efficiency of mineralization of 4C2NP was significantly increased by the UV–Fenton method [2]. Gharbani et al. [3] reported that ozone degraded 4C2NP via the formation of chlorophenol. It was observed that the photochemical oxidation is more efficient than photooxidation or chemical oxidation for removal of 4C2NP [2]. Mehrizad et al. [4] reported the absorption of 4C2NP by single walled and multiwalled carbon nanotubes and suggested that these nanotubes can be used for removal of 4C2NP from the aqueous solution. Although, several physico-chemical methods have been applied for removal of the 4C2NP, these methods are not as successful as microbial degradation [1].

To date, few studies have been reported for microbial degradation of 4C2NP [1]. The microbial degradation of 4C2NP was initiated by either reductive or oxidative mechanism. In the reductive mechanism, 4C2NP may undergo reduction with the formation of 4-chloro-2-aminophenol (4C2AP) [1]. This reaction was catalyzed by either an oxygen sensitive reductase under anaerobic condition or an oxygen insensitive reductase under aerobic conditions [1]. In the oxidative mechanism, the degradation of 4C2NP was catalyzed by an oxygenase with release of nitrite ion. This reaction was observed under aerobic condition. The first evidence of microbial degradation of 4C2NP was observed by a genetically engineered bacterium, *Pseudomonas* sp. N31 that degraded 4C2NP via formation of chlorocatechol and release of nitrite and chloride ions [5]. Beunink and Rehm [6] reported the complete mineralization of 4C2NP by a mixed coculture of *Enterobacter cloacae* and an *Alcaligenes* sp. TK-2. *E. cloacae* biotransformed 4C2NP to 4C2AP under anaerobic conditions that was further degraded aerobically by *Alcaligenes* TK-2 with release of ammonium ions [6]. Arora and Jain [7] reported detoxification of 4C2NP via the formation of 4C2AP, 4-Chloro-2-acetaminophenol (4C2AAP) and 5-chloro-2-methylbenzoxazole (5C2MBZ) by a marine *Bacillus* sp. MW-1.

The aims of this study are (i) to isolate a bacterium that can degrade high concentration of 4C2NP (1.0 mM or above) and (ii) study of metabolic pathway of 4C2NP.

## Materials and Methods

### Chemicals

4C2NP and 4C2AP were purchased from Aldrich (Milwaukee, Wis.). 4C2AAP was synthesized from 4C2AP as described previously [6]. 5C2MBZ was purchased from Across Organics. All other chemicals were used of high purity grade.

**Figure 1:**
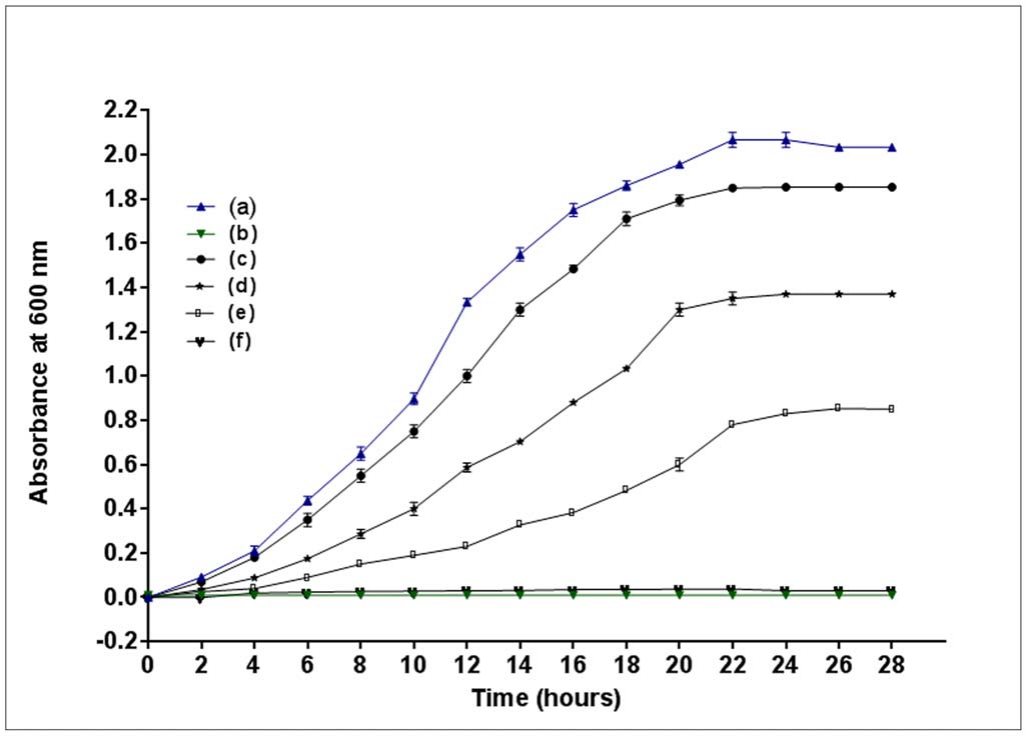
Growth curves of *Bacillus subtilis* RKJ 700 in different conditions. Growth of *Bacillus subtilis* RKJ 700 in (a) 10 mM glucose (as positive control), (b) 0.5 mM 4C2NP (negative control), (c) ) 10 mM glucose and 0.5 mM 4C2NP, (d) ) 10 mM glucose and 1 mM 4C2NP, (e) 10 mM glucose and 1.5 mM 4C2NP, and (f) 10 mM glucose and 2.0 mM 4C2NP.

### Media and Growth Conditions

Minimal media for growth of strain RKJ 700 was prepared by dissolving following compounds in 200 ml distilled water: 0.8 g Na_2_HPO_4_, 0.4 g KH_2_PO_4_, 0.16 g (NH_4_)_2_SO_4_, 0.16 g MgSO_4_.7H_2_O [8]. Trace element solution (200 ml) was added to the solution after above mentioned compounds were dissolved completely. The stock of the trace element solution was prepared by dissolving following compounds in 1 litter distilled water: : 0.10 g Al (OH)_ 3_, 0.05 g SnCl_2_.2H_2_O, 0.05 g KI, 0.05 g LiCl, 0.08 g MgSO_4_, 0.05 g H_3_BO_3_, 0.10 g ZnSO_4_.7H_2_O, 0.01 g CoCl_2_, 0.01 g NiSO_4_.6H_2_O, 0.05 g BaCl_2_, 0.05 g (NH_4_)_6_Mo_7_O_24_.4H_2_O [5]. The pH of minimal media was adjusted to 7.0 before autoclaving at 15 lbs for 20 min. The 50 mM stock solution of 4C2NP was prepared and the desired concentration of the compound was added after filter sterilization (0.22 µm, Millipore). Filter sterilized glucose was added as an additional carbon and energy source at appropriate concentrations. All experiments were performed in 1 liter or 500 ml Erlenmeyer flasks containing 200 ml minimal media, 10 mM glucose and desired concentration of 4C2NP. The flasks were sealed with cotton plugs. After the autoclave, 2% microbial culture of overnight grown cells of strain RKJ 700 was added to the flasks and flasks were incubated at 30°C under the shaking conditions (200 rpm). All experiments were performed in triplicates.

**Figure 2:**
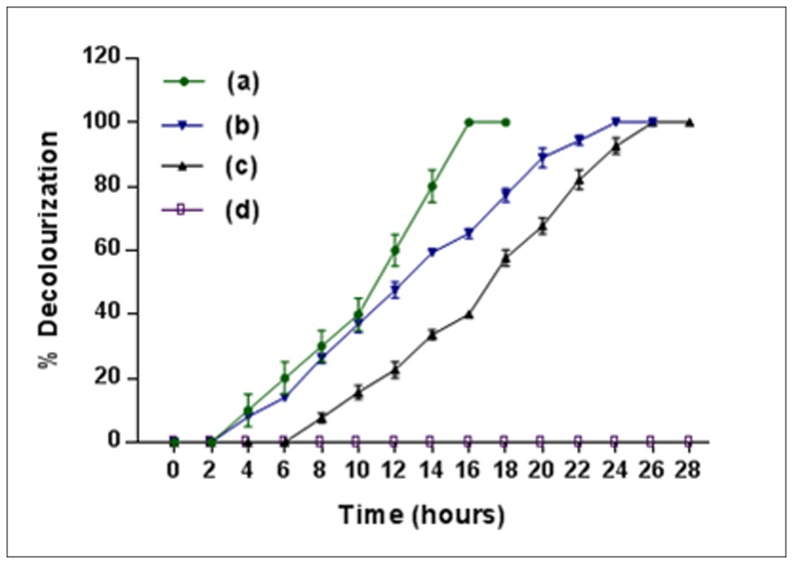
Decolourization of 4C2NP by *Bacillus subtilis* RKJ 700 at various concentrations. Decolourization of the minimal media containing 10 mM glucose and (a) 0.5 mM 4C2NP, (b) 1.0 mM 4C2NP, (c) 1.5 mM 4C2NP, and (d) 2.0 mM 4C2NP.

### Isolation of Bacterial Strain

Soil was collected from a pesticide-contaminated site of Bathinda (30°12′47′′N 74°58′26′′E ), Punjab, India. Bathinda is a highly polluted area of Punjab, India where agriculture soil has been contaminated with several pesticides such as Lindane, Dichlorodiphenyltrichloroethane, Endosulfan, heptachlor, ethion, chlorpyrifos, monocrotophos, chlorpyrifos, malathion and phosphamidon because of their uses [8]. The residues of these pesticides have been detected in the blood samples of peoples collected from three villages of Bathinda district [8].

No specific permits were required for collection the sample from a pesticide contaminated site. I confirm that the location is not privately-owned or protected in any way. I confirm that the field studies did not involve endangered or protected species.

An enrichment method was used to isolate a 4C2NP degrading bacteria from the soil sample. The compound 4C2NP provides a yellow colour to the minimal media when it adds into the medium. The degradation of 4C2NP was monitored by the decolourization of the yellow colour. For the enrichment, 1g soil sample was added to 500-ml flask containing 200 ml minimal medium, 1.0 mM 4C2NP and 10 mM glucose as the additional carbon source. Upon decolourization of flask, cultures were serially diluted and plated on minimal media agar plates containing 1 mM 4C2NP with 10 mM glucose. On the basis of decolourization of the 4C2NP agar plates, one bacterium designated as strain RKJ 700 was selected for the further study.

**Figure 3:**
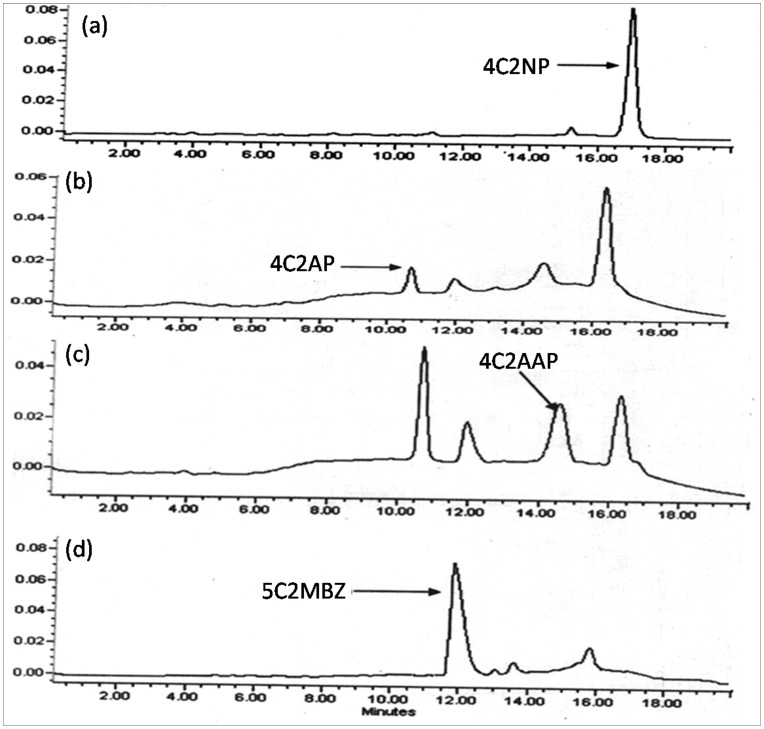
HPLC analysis of the samples collected from different intervals. HPLC elution profiles of the sample collected from (a) 0 h, (b) 8 h, (c) 16 h and (d) 24 h.

**Figure 4:**
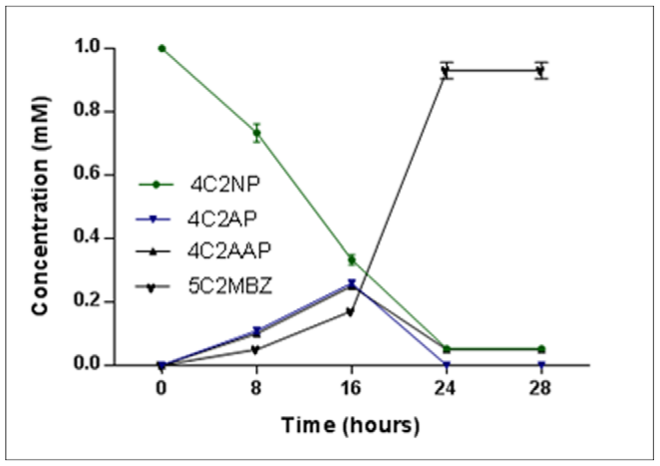
Degradation curve of 4C2NP based on HPLC data. Quantification of amounts of 4C2NP and its metabolites in sample collected from different intervals (0, 8, 16, 24 and 28 h).

**Figure 5:**
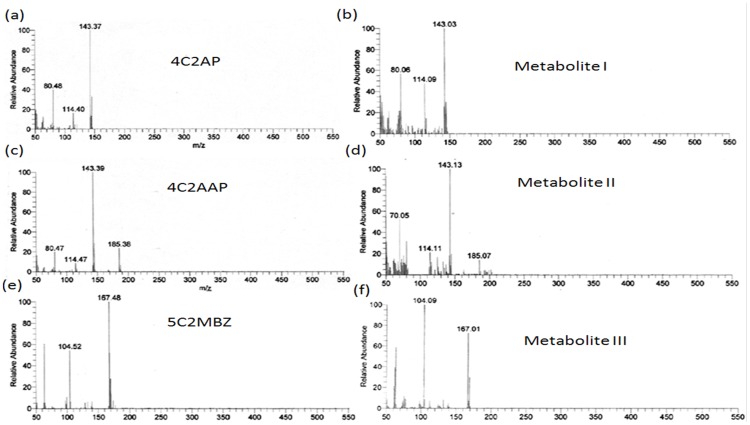
Mass fragments of metabolites and authentic standards. Mass fragments of the authentic 4C2AP (a), metabolite I (b), authentic 4C2AAP (c), metabolite II (d), authentic 5C2MBZ (e) and metabolite III (f).

**Figure 6:**
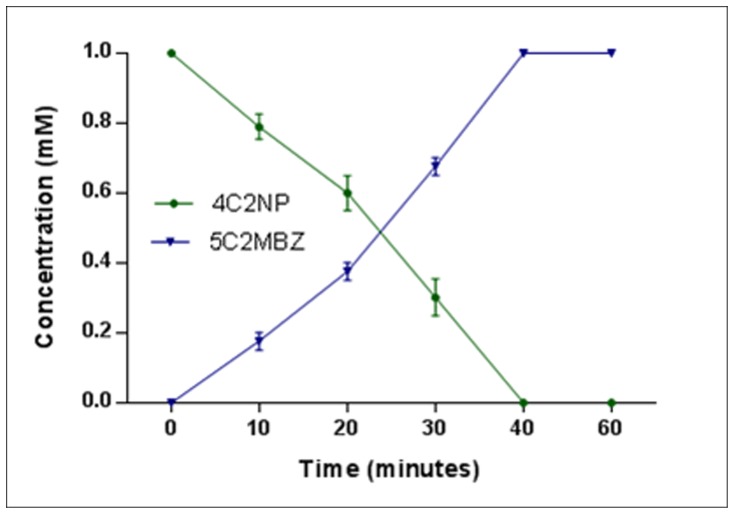
Resting cell studies showing stoichiometric formation of 5C2MBZ from 4C2NP. The resting cells of *Bacillus subtilis* RKJ 700 completely depleted 4C2NP with stoichiometric formation of 5C2MBZ within 40 minutes.

**Figure 7:**
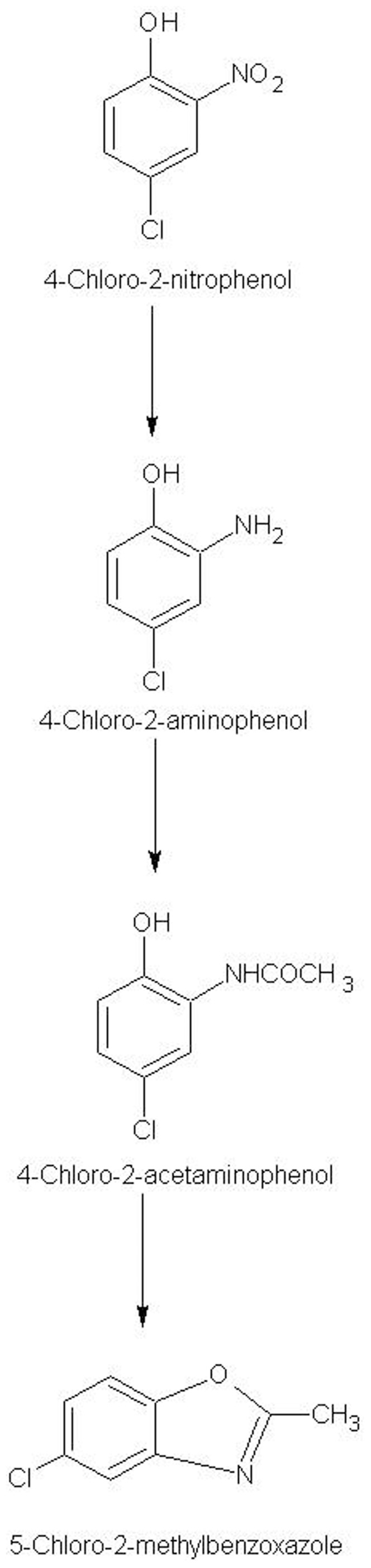
Proposed pathway of degradation 4C2NP for *Bacillus subtilis* RKJ 700. 4C2NP initially reduced to 4C2AP, which undergo acetylation to form 4C2AAP. After cyclization, 4C2AAP forms 5C2MBZ.

### Identification of 4C2NP Decolourizing Strain

Strain RKJ 700 was identified on the basis of 16S rRNA gene sequencing. The 16S rRNA gene was amplified by PCR using the universal primers 8-27F (5′-AGAGTTTGATCCTGGCTCAG-3′) and 1492R (5′-TACGGYTACCTTGTTACGACTT-3′) as described previously [9], [10], [11], [12]. The PCR amplification reaction mix (25 µl) contained 50–100 ng of genomic DNA, 2.5 µl of 10 X Taq polymerase buffer, 200 µM of each dNTP, 1.0 U of *Taq* DNA polymerase (New England Biolabs, MA, USA), 20 pmol of each primer (BioBasic Inc. Ontario, Canada) and water. Amplification was carried out using a personal thermocycler (Eppendorf, Hamburg, Germany) [9], [10], [11], [12]. Amplification program consisted of an initial denaturation at 94°C for 3 min followed by 30 cycles of denaturation at 94°C for 1 min, annealing at 55°C for 1 min, extension at 70°C for 1 min, and final extension at 72°C for 5 min [9], [10], [11], [12]. The amplified PCR product was sequenced using Big Dye terminator cycle sequencing ready reaction kit (Applied Biosystems) by an automated DNA sequencer (ABI 3130 XL Genetic Analyzer; Applied Biosystems) [8], [9], [10], [11]. The 16S rRNA gene sequence similarity of strain RKJ 700 was determined by using BLAST.

### Growth and Decolourization Studies

To monitor the effects of different concentrations of 4C2NP on the growth on strain RKJ 700 and decolourization of 4C2NP, strain RKJ 700 was grown on 1 liter Erlenmeyer flask containing 200 ml minimal media, 10 mM glucose and appropriate concentration of 4C2NP (0.5 mM/1.0 mM/1.5 mM/2.0 mM). Samples were collected at regular intervals and the bacteria growth was monitored by taking absorbance at 600 nm.

For decolourization studies, the samples collected at regular intervals were centrifuged. The decolourization was monitored by the measuring the absorbance of supernatant at 430 nm using U.V-Visible spectrophotometer. The percentage decolourization was calculated according to the formula:

% Decolourization = (Initial Absorbance − Absorbance after time t)×100/Initial Absorbance.

### Identification of Metabolites

Strain RKJ 700 was grown in 1 liter Erlenmeyer flask containing 200 ml minimal media, 1.0 mM 4C2NP and 10 mM glucose. Samples (50 ml) were collected at regular intervals and centrifuged at 8000 g for 15 min. The samples were extracted with ethyl acetate and analyzed by high performance liquid chromatography (HPLC) and gas chromatography-mass spectrometry (GC-MS) as described previously [7], [9].

HPLC analysis was carried out using a Waters 600 model high performance liquid chromatography equipped with a photodiode array detector system [7], [9]. The compounds were separated on a C_18_ reverse-phase silica column using 1% glacial acetic acid in methanol and 1% glacial acetic acid in HPLC grade water at a ratio of 80∶20 as the mobile phase. Flow rate was 1.0 ml/min; injection volume was 15 µl, and the compounds were detected at 280 nm and 300 nm [7], [9].

GC-MS analysis was carried out using a GC-MS-QP5000 instrument (Shimadzu, Tokyo, Japan) equipped with quadrupole mass filter and DB-1 capillary column with ionization of 70 eV, scan interval 1.5 s and mass range of 50–550 Da [7], [9]. The column temperature was initially increased from 80°C to 160°C at the rate of 5°C min^−1^ and then from 160°C to 260°C at the rate of 10°C min^−1^. The carrier gas (Nitrogen) flow rate was 20 ml min^−1^.

### Resting Cell Studies

Cells of strain RKJ 700 were grown on 1 liter Erlenmeyer flask containing 10 mM glucose and 1 mM 4C2NP. The flask was incubated at 30°C under shaking conditions (200 rpm). Cells were centrifuged in 250 ml centrifuged bottles with sealing caps (NALGENE), just prior to the decolourization, and washed with ice-cold phosphate buffer (10 mM, pH 7.2) and re-suspended in 20 ml of the same buffer [7]. The 4C2NP was added as filter sterilized solution at a final concentration of 1.0 mM and centrifuged bottles were incubated at 30°C under shaking conditions (120 rpm). The samples (1.0 ml) were removed at different time points [7]. The collected samples were immediately centrifuged at 13,000 rpm at 30°C for 2 min and supernatants were filtered through 0.2 µm filter (Millipore) and analyzed by HPLC. The quantification of substrate and products was done with HPLC using standard curve(s) prepared with authentic standard(s) [7]. In the control experiment, non-induced cells of strain RKJ 700 grown on 10 mM glucose alone were used and the remaining process is same as described above.

### Detection of Ammonia, Nitrite and Chloride

For detection of the ammonia, nitrite and chloride ions in the medium, 3 ml samples were collected at regular intervals and centrifuged at 13,000 rpm for 5 min. The supernatant was divided into 3 aliquots and subjected to test for the release of ammonium, nitrite and chloride ions, respectively. Non-inoculated samples were taken as appropriate negative control.

Ammonia was detected by measuring the oxidation of NADH in the presence of 2-oxoglutarate and L-glutamate dehydrogenase using ammonia estimation kit purchased from Sigma (USA) following the manufacturer’s instruction.

Release of chloride ion was detected and quantified following a colorimetric method [7]. In brief, 400 µl of culture supernatant(s) was diluted with distilled water up-to total volume of 2.0 ml [7]. To the diluted sample, 200 µl each of 250 mM ferric ammonium sulphate (prepared in 9N nitric acid) and saturated solution of mercuric-thiocyanate (prepared in ethanol) were added [7], mixed properly and the reaction was allowed to proceed at room temperature for 10–15 min. If chloride ions are present in the sample, pink color is observed which show absorbance at 460 nm [7].

Nitrite ions were detected as described previously [9]. For nitrite release, 200 µl of reagent A [0.1% (w/v) sulfanilic acid (Merck) in 30% (v/v) acetic acid] was added into the 200 µl of culture supernatant and mixed properly [9]. After 1 min, 200 µl of reagent B [0.1% (w/v) *N*-(1-naphthyl)-ethylenediamine dihydrochloride (Sigma) in 30% (v/v) acetic acid] was added and incubated at room temperature for 15 min. Presence of nitrite ion in the sample was indicated by the appearance of purple colour and quantified by calculating the absorbance at 540 nm [9].

## Results

### Isolation and Identification of Bacterial Strain

A 4C2NP decolourizing bacterial strain RKJ 700 was isolated from soil collected from a pesticide contaminated site of Punjab, India. Strain RKJ 700 was identified as *Bacillus subtilis* based on the 16S rRNA gene sequence analysis. The 16S rRNA gene sequence of strain RKJ 700 was deposited at the Genbank, (National Center for Biotechnology Information) under accession number HM027881.

### Growth and Degradation Studies

The 4C2NP is a toxic substance and suppresses the growth of the several microorganisms. I have monitored the effect of different concentrations of 4C2NP on the growth of *Bacillus subtilis* RKJ 700 in the presence of additional carbon source, i.e., glucose (Figure 1). It was observed that *Bacillus subtilis* RKJ 700 was able to grow in the presence of 4C2NP up to concentration of 1.5 mM. The optical density of strain RKJ 700 was decreased with increase the concentration of 4C2NP. No bacterial growth was observed when the concentration of 4C2NP was 2.0 mM.

I have also investigated the effect of different concentrations of substrate on the 4C2NP decolourization by *Bacillus subtilis* RKJ 700 (Figure 2). Strain RKJ 700 decolourized 4C2NP up to a concentration of 1.5 mM. There was no decolourization at 2.0 mM concentration due to the inhibition of the growth of the cells of strain RKJ 700. It was observed that decolourization of 4C2NP was faster at low concentration (0.5 mM) as compare to high concentration (1.5 mM). Strain RKJ 700 decolorized 1.0 mM 4C2NP completely within 24 hours.

### High Performance Liquid Chromatography (HPLC) Analysis

HPLC studies confirmed the complete depletion of 4C2NP with appearance of three metabolites (Figure 3). In the sample of the 0 h, only parent compound (4C2NP) was detected. In the sample of 8 h and 16 h, metabolites I, II and III were detected and their retention times were 10.69 min, 14.43 min and 11.74 min, respectively. In the sample of 24 h, metabolites III was detected with disappearance of the metabolite I and II, indicating their conversion into metabolite III. The retention times of these metabolites (metabolite I, II and III) were exactly matched with authentic standards of 4C2AP, 4C2AAP and 5C2MBZ, respectively. Degradation curve based on the HPLC data showed that 4C2NP was degraded within 24 hours with stoichiometric formation of metabolite III [Figure 4].

### Gas Chromatography-Mass Spectrometry (GC-MS) Analysis

To confirm the identity of these metabolites, GC-MS studies were carried out. The mass fragment of metabolites I, II and III were 143 m/z, 185 m/z and 167 m/z that were exactly matched to that to authentic 4C2AP, 4C2AAP and 5C2MBZ, respectively (Figure 5).

On the basis of GC-MS analysis, metabolites I, II and III were identified as 4C2AP, 4C2AAP and 5C2MBZ, respectively. 4C2AP was a first metabolite of degradation pathway of 4C2NP, which may be formed directly from 4C2NP via reduction of nitro group to amino group, which has been identified as initial step of degradation of 4C2NP [6], [7]. The acetylation of amino group of 4C2AP may cause the formation of 4C2AAP (metabolite II) which may form 5C2MBZ (metabolite III) after cyclization [7].

### Resting Cell Studies

Resting cells of strain RKJ 700 decolourized 1 mM 4C2NP within 40 minutes whereas no decolourization was observed in non-induced control cells at the same time. These data suggest that enzyme system catalyzing transformation is inducible in *Bacillus subtilis* RKJ 700. Furthermore, HPLC analysis of the samples collected from resting cells of strain RKJ 700 confirmed the complete depletion of 4C2NP with stoichiometric formation of 5C2MBZ (Figure 6).

### Ammonia, Nitrite and Chloride Releases

No chloride, ammonium and nitrite ions were detected from 4C2NP by *Bacillus subtilis* RKJ 700 during whole transformation process.

## Discussion

A 2C4NP degrading bacteria, *Bacillus subtilis* RKJ 700 was isolated from soil sample collected from a pesticide-contaminated site, Punjab, India. Strain RKJ 700 decolourized 4C2NP only in the presence of additional carbon source. The unique feature of strain RKJ 700 is that *Bacillus subtilis* RKJ 700 was able to decolourize 4C2NP up to a concentration of 1.5 mM.

My studies clearly showed that *Bacillus subtilis* RKJ 700 decolourized 4C2NP via the formation of 4C2AP, 4C2AAP and 5C2MBZ. It has been suggested that 4C2NP was initially reduced to 4C2AP, which further acetylated into 4C2AAP. The acetylated product 4C2AAP was transformed to 5C2MBZ, which was detected as a final product in the biotransformation pathway of 4C2NP (Fig. 7). This biotransformation pathway was first observed in a marine bacterium, *Bacillus* sp. MW-1, which was able to transform 4C2NP up to concentration of 0.3 mM [4]. I have observed similar mechanism in a soil bacterium, *Bacillus subtilis* RKJ 700, which decolourized 4C2NP up to concentration of 1.5 mM. This is the first report of degradation of 4C2NP at high concentration (1.5 mM).

The degradation of 4C2NP was initiated with formation 4C2AP, which was 500 fold less toxic than 4C2NP [7], [13]. Generally, it has supposed that amino derivatives are 500 fold less to toxic than their corresponding nitro derivatives due to the conversion of an electron-withdrawing nitro group to an electron donating amino group [13]. Furthermore, due to presence of electron-donating amino group, the aromatic compound becomes more susceptible for microbial degradation via electrophilic attack [7]. The formation of 4C2AP was also observed in degradation studies of 4C2NP by a mixed-coculture of *Enterobacter cloacae* and an *Alcaligenes* sp. TK-2 [6]. *Enterobacter cloacae* biotransformed 4C2NP to 4C2AP, which was further degraded by *Alcaligenes* sp. TK-2 with release of ammonia ions [6]. No ammonium ions were detected during the degradation of 4C2NP by *Bacillus subtilis* RKJ 700 suggesting that the further degradation of 4C2AP was proceeded without ammonia release from 4C2AP. The direct ring cleavage of 4C2AP was also reported and catalyzed by the enzyme, 2-aminophenol-1,6-dioxygenase, which is a key mechanism when bacteria utilized a chlorinated nitroaromatic compound as a sole carbon and energy source and 4C2AP was formed as a intermediate [14]. *Bacillus subtilis* RKJ 700 did not utilize 4C2NP as the sole carbon and energy source, therefore the direct cleavage to 4C2AP may not be involved in the degradation pathway of 4C2NP in strain RKJ 700.

Another mechanism of degradation of 4C2AP is acetylation, which has been considered as a detoxification mechanism in a number of bacteria [15], [16], [17], [18]. The acetylation of 4C2AP was first reported in the degradation pathway of 3-chloronitrobenzene and 4C2AAP was identified as acetylated product, which was further degraded with release of chloride and ammonia [15]. Arora and Jain [7] also detected acetylation of 4C2AP into 4C2AAP in the degradation of 4C2NP by a marine bacterium, *Bacillus* sp. MW-1; however acetylated product 4C2APP was further transformed into a complex compound 5C2MBZ without release of chloride and ammonium [7]. In this study, I have also been reported the formation of 5C2MBZ from 4C2NP by *Bacillus subtilis* RKJ 700.

The mechanism of degradation of 4C2NP was reductive in *Bacillus subtilis* RKJ 700, *Bacillus* sp. MW-1 and a mixed-coculture of *Enterobacter cloacae* and an *Alcaligenes* sp. TK-2 [1]. However, the oxidative pathway of the degradation of 4C2NP has been observed in a genetically engineered bacterium, *Pseudomonas* sp. N31 that degraded 4C2NP with release of chloride and nitrite ions and formation of chlorocatechol [5].

The studies on transformation of nitroaromatic compounds by *Bacillus* spp. are relatively rare; however, few recent studies have been successfully reported with the abilities of *Bacillus* spp. to transform nitroaromatic compounds. Perreault et al. [19] reported biotransformation of 2,4-dinitroanisole by a *Bacillus* sp. G-12, which degraded 2,4-dinitroanisole via reduction and further acetylation. The degradation of 2,4-dinitroanisole was initiated with reduction of nitro group at *ortho*-position and as a result, 2-amino-4-dinitroanisole was formed which was acetylated to 2-acetylamino-4-dinitroanisole [19]. The initial mechanism of biotransformation of 2,4-dinitroanisole was similar to biotransformation of 4C2NP by *Bacillus* sp. MW-1 and *Bacillus subtilis* RKJ 700 where the degradation of 2C4NP was also initiated with reduction and acetylation mechanism [19]. These data suggest that *Bacillus* spp. may have similar mechanism for biotransformation of nitroaromatic compounds.

Recent studies showed that several researchers have engaged with their studies on the biodegradation of various pesticides, chloronitrophenols and other nitroaromatic compound [1], [7], [9], [20]. A few strains of *Bacillus subtilis* have been characterized with their abilities to degrade various pesticides and aromatic compounds [21], [22], [23], [24]. Zessi et al. [21] reported that *Bacillus subtilis* degraded p-aminoazobenzene to aniline and *p*-phenylinediamine in the presence of glucose under aerobic conditions. Das and Mukherjee [22] studied the degradation of crude-petroleum-oil hydrocarbons by *Bacillus subtilis* DM-04. Zang et al. [23] reported the degradation of 2-naphthol by coupling of *Aspergilius niger* and *Bacillus subtilis*. Initially the fungus *Aspergillus niger* biotransformed 2-naphthol to 1,2-naphthalene-diol and 1,2-naphthoquinone, which were utilized by *Bacillus subtilis* as the sole carbon and energy sources. Myresiotis et al. [24] studied the degradation of five pesticides acibenzolar-S-methyl, metribuzin, napropamide, propamocarb hydrochloride and thiamethoxam by *Bacillus subtilis* GB03, *Bacillus subtilis* FZB24, *Bacillus amyloliquefaciens* IN937a and *Bacillus pumilus* SE34 in liquid culture as well as soil microcosm [24]. These data indicate that strains of *Bacillus subtilis* have ability to degrade a variety of xenobiotic compounds. The genetic analysis of degradation of various xenobiotic compounds by *Bacillus* spp. is subject of further research.

### Conclusion

A soil bacterium, *Bacillus subtilis* RKJ 700 decolourized high concentration of 4C2NP and transformed it into 5C2MBZ. This is the first report of (i) the degradation of 4C2NP at high concentration (1.5 mM), and (ii) the formation of 5C2MBZ by a soil bacterium.
